# Deep Learning-Based Fake Information Detection and Influence Evaluation

**DOI:** 10.1155/2022/8514430

**Published:** 2022-02-23

**Authors:** Ning Xiang

**Affiliations:** School of Journalism and Communication, Hunan Mass Media Vocational and Technical College, Changsha 410100, China

## Abstract

With the prevalence of the Internet, a large number of users have participated in OSN (Online Social Networks), which has gradually made it the mainstream way for obtaining news or information from the Internet. However, with the rapid development of the Internet, a large amount of fake information has also been spread on the Internet. Therefore, fake information detection is of great significance at the moment. A multimodal fake information detection method is proposed in this article, which has adopted the textual and visual contents in the piece of information to make the judgments. The textual feature representation vector is firstly obtained through the pretraining of the Bert model, and then the visual feature representation is obtained through the pretraining of the VGG-19 model. From the proposed method, two MCBP (Multimodal Compact Bilinear Pooling) modules are adopted. The first MCBP module is adopted to obtain the visual feature representation vector with attention, and the second MCBP module is adopted to join the visual feature with the attention mechanism and the textual feature vector. Then, the joined vector can be adopted for fake information detection. The proposed method in this article is compared with two baseline methods. The experimental results on the Twitter and Weibo datasets have proved that the proposed method in this article is better than the EANN method and the SpotFake method in terms of accuracy, precision, recall, and F1 score.

## 1. Introduction

With the popularity of the Internet, a large number of users have participated in OSN (Online Social Networks), which has gradually made it the mainstream method for obtaining news or information from the Internet. However, with the rapid development of the Internet, much fake information was also spread on the Internet. In particular, the creation of the programmed social accounts, called Socialbots [1–3], has flooded the OSN with information generated from Socialbots. According to a report by the company GlobalDots, the traffic generated by Socialbots on social platforms in 2018 has reached about 37% of the total traffic, of which malicious traffic accounted for about 20% [4]. From the overall traffic, we can see the severity of fake information. On the other hand, the propagation of some fake information has seriously impacted society, which can even affect political elections and manipulate global stock markets. For example, in 2016, the fake news storm has affected the US election. In 2017, a large amount of spam information was generated by Socialbots during the election of the German Chancellor. Also, in 2017, a large amount of fake information was generated by Socialbots just before the French presidential election [5–7]. In addition, with the spread of the new COVID epidemic, all kinds of fake information are also flooded on the Internet, which has a serious impact on the lives of the public. Therefore, fake information detection is an important topic at present, and blocking the spread of fake information is of great significance to the normal operation of society.

The methods of fake information detection can be described mainly from two aspects, the first is the relevant features for fake information detection, and the second denotes the model. Overall, the features for fake information detection can be divided into two types: one denotes the features from the content and the other denotes the contextual feature. In the method of content-based fake information detection, it is performed by extracting relevant features in fake information, such as textual features and visual features [8–10]. Textual features generally consisted of word frequency, special vocabulary, total number of words, and syntactic features. Visual features often include sharpness, histogram features, and consistency features. The difference between the contextual features and the content features is that the contextual features are not limited to the current piece of information. In fact, the contextual features can describe the information within the propagation of the information. Normally, it includes the following aspects. On the user characteristic level, it includes the information forwarded by the user, including the user's attention and the user's registration time. The other is within the process when the user forwards the information, including the stance network, friend network, forwarding time, and forwarding group. As the fake information detection methods based on contextual features involve the propagation model and require a larger amount of data, it will not be studied in this article. The fake information detection methods herein only include content-based detection methods; that is, only the content for this piece of information is adopted for detection.

The features for content-based fake information detection can usually be divided into textual features and visual features. The extraction of textual features and visual features are introduced as follows. The representation methods of textual features include the bag-of-words method [11, 12], which treats the text as a collection of independent vocabulary, ignoring the grammatical and word sequences in the text. For the TF-IDF method [13, 14], the importance of different vocabulary to the overall text is considered. It is assumed that the importance of the vocabulary is proportional to the frequency of appearance in the text and inversely proportional to the frequency of appearance in the corpus. In the Word2Vec method [15, 16], the low-dimensional word embedding can be learned through a shallow neural network according to the corpus. The Word2Vec method includes the CBOW model and the skip-gram model. In the skip-gram model, the importance of words that are closer is greater than those farther away, which is different from the CBOW model. In practical application, the Word2Vec method is usually adopted as a pretraining method to generate the corresponding word embedding vector. In this representation, the underlying information in the existing corpus can be fully utilized. In this condition, words with similar semantics also have a closer metric in the word embedding vector space. The Bert model [17, 18] is transformer-based and is a deep, bidirectional, and unsupervised representation model. This model can sufficiently adapt contextual information. After training through a large number of the corpus, ambiguities can be weakened compared to methods such as Word2Vec. At present, the Bert model has the best representation capability. For visual vector representation, it is currently relatively mature, including ResNet [19], VGGNet [20] with repeated modules, GoogleLeNet [21], which is a fusion of CCN and RNN models, and AlexNet [22]. These models are relatively mature, so they are not repeated here.

Content-based fake information detection methods can be divided into single-modal-based methods and multimodal methods according to the types of content adopted. Among the single-modal-based methods, only a single source of information, such as text information or visual information, is adopted for detection. In the multimodal-based methods, multiple types of information are joined for comprehensive detection. For example, texts and images are adopted for detection. Generally speaking, multimodal-based detection methods have higher accuracy than single-modal-based. For the single-modal-based methods, the authors in [23] proposed to adopt the CNN (Convolutional Neural Network) to learn the deep features in the information to identify true or false. The authors in [24] proposed to detect the authenticity of information through a RNN (Recurrent Neural Network). In [25], by adopting the rich information from the image, fake information detection is performed through a multiregion neural network. In single-modal-based methods, since just a single type of information is adopted, the accuracy can be improved. In multimodal-based methods, textual representation vectors and visual representation vectors can be concatenated to obtain a hybrid representation vector for fake information detection. In the method proposed by Jin et al. [26], an attention-based RNN model is adopted to fuse textual features and visual features, and good detection results can be achieved. In literature [27], an adversarial network is adopted to obtain a multimodal feature extractor so as to eliminate the influence of common features in different information on detection, which can improve the generalization ability of the proposed model. This method can be called the EANN method, where direct concatenation is still applied to fuse the visual and textual feature vectors. The SpotFake model is proposed in literature [28], where the textual feature vector is extracted through a pretrained Bert model, and the visual feature vector is extracted through the pretrained VGG-19 model. Then the two vectors are concatenated to obtain a new feature vector for detection. The SpotFake + model is proposed in literature [29]. Compared with the SpotFake model, it has adopted the improved Bert model XLNet to extract visual feature vectors, which can improve the detection performance compared to the SpotFake method. The authors in [30] have proposed the SAME method, where sentiment classification results are adopted for fake information detection.

A multimodal-based fake information detection method is proposed in this article, which adopts the textual information and visual information from the piece of information to make judgments. Firstly, the textual feature representation vector is obtained through the pretraining of the Bert model, and then the visual feature representation of the image can be obtained through the pretraining of the VGG-19 model. In order to better fuse the two different types of features, instead of simple concatenation, Multimodal Compact Bilinear Pooling (MCBP) is adopted for fusing features in the two different domains. The proposed method in this article is inspired by the Visual Question Answering (VQA) method in literature [31]. The MCBP module is used twice here. The first MCBP module is adopted to obtain the image feature representation with attention, and the second MCBP module is adopted to join the image feature with the attention mechanism and the textual feature vector. And then, fake information detection is performed adopting the joined vectors. The MCBP module adopted in this article can fully integrate the feature vectors of different domains, thereby reducing information loss, at the same time achieving a better computational burden and better backpropagation characteristics. Two OSN datasets, including the Twitter dataset and the Weibo dataset, are adopted to verify the proposed method. Compared with the direct concatenation-based SpotFake method and the EANN method, the proposed method can achieve better accuracy, precision, recall, and F1 score, which has proved the effectiveness of the proposed method.

## 2. Methods

**Figure 1:**
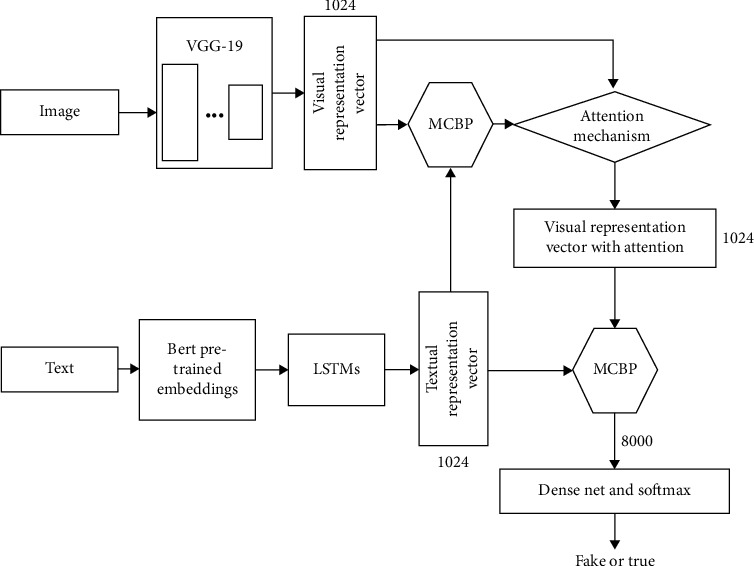
The overall structure of the proposed fake information detection method.

The proposed method in this article is shown in Figure 1. Firstly, the representation vector of the text information is obtained through pretraining from adopting the Bert model. The representation vector is used as the inputs of two different subnetworks. The first subnetwork is an image feature extraction network based on attention, and the second subnetwork is a feature fusion network of different domains. In the attention-based image feature extraction subnetwork, the MCBP module is adopted for the first time to fuse the visual features and the textual features to obtain the attention vector, which can reweight the visual features directly extracted through VGG-19, thereby obtaining the visual feature vector with attention. This attention-based feature and the representation vector of the textual domain based on Bert are then fused in the feature fusion subnetwork through a second MCBP module. The fused feature vector can then pass through the fake information detection subnetwork. Note that the MCBP module is used twice in the proposed method. Both MCBP modules are adopted for feature fusion of the representation vectors from different domains. The first MCBP is adopted to obtain the image representation vector with attention. The second MCBP is to get the feature vector after fusion. The length of the visual and textual representation vector can be regarded as hyperparameters. The hyperparameters are studied in the experiment section. Herein, the dimension of the textual and visual vector is 1024 and the flattened vector after the second MCBP module has a length of 8000. The following describes the proposed method from the following four aspects.

### 2.1. Textual Feature Extraction

**Figure 2:**
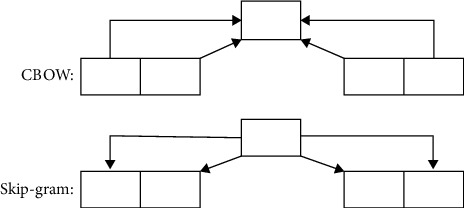
Schematic diagram of CBOW model and skip-gram model.

Among the many textual feature extraction methods, the most typical one denotes the bag-of-words method, where the texts are treated as a collection of independent words, ignoring the grammatical and sequence features in the text. In the TF-IDF method, for a word in a specific document *w*_*i*_, the degree of importance can be expressed as follows:

(1) $$\begin{array}{c} tf_{ij}=\frac{n_{ij}}{\sum_{k}n_{kj}}, \end{array}$$

where *n*_*ij*_ denotes the number of times the word appears in the document *d*_*j*_ and *k* represents the total number of words in the document *d*_*j*_. In this method, the importance of words is considered to be proportional to the frequency of appearance in the text and inversely proportional to the frequency of appearance in the corpus. The classic Word2Vec method, as shown in Figure 2, includes the classic CBOW model and the skip-gram model. Noting that the figure herein is just an illustration figure, which shows the most significant differences between the CBOW and the skip-gram model. For the CBOW model, the contextual words are adopted to predict the central word, while for the skip-gram model, the central word is adopted to predict the contextual word.

**Figure 3:**
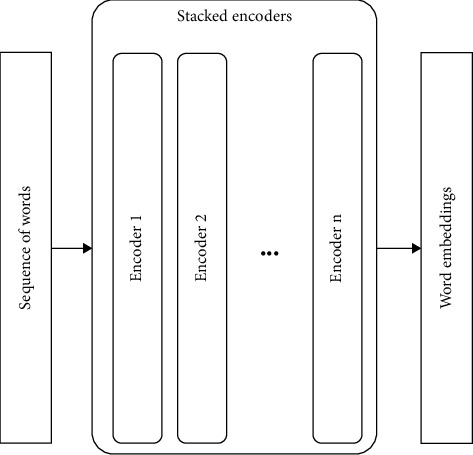
An illustration of the Bert-based textual feature extractor.

In this article, the Bert model is adopted to generate word embedding vectors. As shown in Figure 3, the Bert model in this article is similar to that in [28], with several transformer blocks in the middle, using the words of the moving window as input. The attention mechanism is added to each layer's transformer blocks, and then the attention vector is passed to the transformer block of the next layer.

After the word embedding vectors are obtained by the Bert model, the sequence of the word embedding vectors is passed through a two-layer LSTM layer, where the representation vector of the text sequence can then be obtained.

### 2.2. VGG-19 Model-Based Visual Feature Extraction

In this article, the pretrained VGG-19 neural network by ImageNet dataset is adopted to extract the visual feature vector. Specifically, similar to literature [28], using the output of the last two layers of the classic VGG-19 neural network, and by adding a fully connected layer, we can get the following:

(2) $$\begin{array}{c} V_{\text{vec}}=\sigma \left(W_{\text{dense}}V_{\text{VGG}-2}\right). \end{array}$$

Among them, *W*_dense_ represents the weight of the fully connected layer, and *V*_vec_ represents the output of the fully connected layer, which is adopted as a feature vector extracted from the image for later processing.

### 2.3. The MCBP Module

When there exists a multimodal feature vector fusion, especially when the feature vectors are extracted from different domains, only element-wise product or sum of two feature vectors, or by direct catenation, will often lead to the deterioration of the final recognition rate due to loss of relevant information of the features in different domains. In the fusion of multimodal-based textual and visual feature fusion, bilinear pooling is a rather reasonable choice. The two feature representations can be fused through the outer product, so that the fused feature representation is translationally invariant. Assuming that two features A and B can be represented as *f*_*A*_(*l*, *I*) ∈ *R*^*L*×*M*^ and *f*_*B*_(*l*, *I*) ∈ *R*^*L*×*N*^, respectively, where *l* represents the position in the image, and *L* × *M* and *L* × *N*, respectively, represent the dimension of the feature, then bilinear pooling can be performed as follows:

(3) $$\begin{array}{c} b\left(l,I,f_{A},f_{B}\right)=f_{A}{\left(l,I\right)}^{T}f_{B}{\left(l,I\right)}^{T}\\ \zeta \left(l\right)=\sum_{l}b\left(l,I,f_{A},f_{B}\right),\\ x=\text{flatten}\left(\zeta \left(I\right)\right),\\ y=\text{sign}\left(x\right)\sqrt{\left|x\right|},\\ y=\frac{y}{{\left\|y\right\|}_{2}}. \end{array}$$

The function flatten(.) means transforming the matrix into a vector, which is denoted as *x*. The flattened vector is then normalized to obtain the fused feature *y*. In this article, due to the reason that the related feature dimensions are relatively high and that the bilinear pooling are used twice, the overall computational burden is relatively large. Therefore, direct use of bilinear pooling is not suitable for the application of this article.

**Figure 4:**
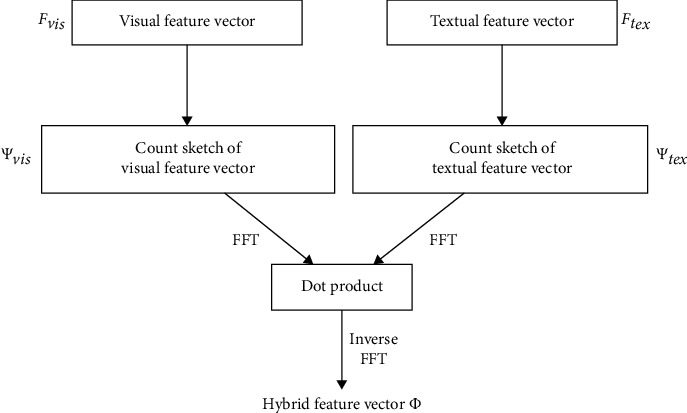
The basic process of the MCBP module.

In this article, a new method for fusing two features from different domains is adopted, which has adopted the MCBP module [31]. The basic process of the proposed method is shown in Figure 4. Supposing that the visual feature vector is expressed as *V*_*a*_ ∈ *R*^*n*^ and the textual feature vector is expressed as *V*_*b*_ ∈ *R*^*m*^, the structure is shown as follows.

Compared with the previous direct bilinear pooling method, this module has projected the outer product into a low-dimensional space through the count sketch projection function. The projection process adopting the count sketch projection function is shown as follows, which has projected the vector *V*_*a*_ to *V*_*b*_. Firstly, two vectors are initialized, where

(4) $$\begin{array}{c} s\in \left\{1,-1\right\},\\ h\in {\left\{1,2,3,\ldots ,m\right\}}^{n}. \end{array}$$

It means that the element in *s* is either 1 or -1, and *h* means that the index *i* in the initial vector *V*_*a*_ is projected to the index *j* in the vector *V*_*b*_. Among them, *s* and *h* are randomly selected from the uniform distribution. The update process is shown as follows, where the index *i* is from 1 to n:

(5) $$\begin{array}{c} V_{a}\left[i\right]j=h\left[i\right]s\left[i\right]V_{a}\left[i\right]V_{b}\left[j\right],\\ V_{b}\left[h\left[i\right]\right]=V_{b}\left[h\left[i\right]\right]+s\left[i\right]V_{a}\left[i\right]. \end{array}$$

Suppose that *V*_*b*_ is initially set as a zero vector. Then according to the above updating formula, in the resulting projecting vector *V*_*b*_, its corresponding destination index can be expressed as *j*=*h*[*i*], and then *s*[*i*]*V*_*a*_[*i*] is added to *V*_*b*_[*j*].

After the dimensionality reduction projection, the two vectors *F*_vis_ and *F*_tex_ are subjected to a convolution operation. In order to speed up the convolution operation, it can be converted into a combination of forward and inverse FFT transformations:

(6) $$\begin{array}{c} \Phi =FFT^{-1}\left(FFT\left(F_{\text{vis}}\right)\cdot FFT\left(F_{\text{tex}}\right)\right). \end{array}$$

Among them, the operation of · represents element-wise multiplication.

## 3. Experimental Results

In order to verify the effectiveness of the proposed method in this article on fake information detection, two different datasets are adopted herein. Both datasets are from the OSN platform: one is the Twitter dataset [32], and the other is the Weibo dataset [26]. Among them, the Twitter dataset consists of a collection of tweets from 17 events and about 2000 other tweets. In the Weibo dataset, all information is manually labeled as true and fake, with the fake information deemed true and already spread on the network. In both the two datasets, the true and fake information is marked, and the detailed statistics of the two datasets are listed in Table 1. In this article, all data samples are divided into training set and test set with a 70%–30% split.

### 3.1. The Effects of Different Hyperparameters

**Figure 5:**
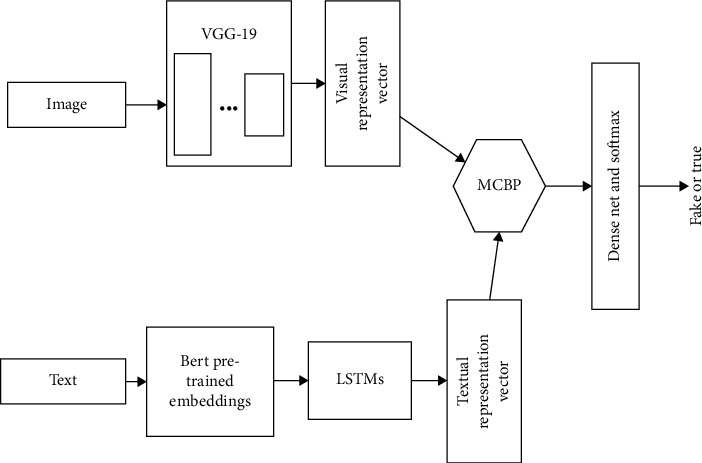
The structure of the single-MCBP based method.

Two MCBP modules are adopted in the proposed method. The first MCBP module has added an attention mechanism to the visual feature vector, and the second MCBP module integrates visual features and textual features.  Table 2 gives the accuracy, precision, recall, and F1 score comparisons under different datasets and under different network settings. Note that here the two different network settings herein are as follows: (1) the proposed structure with two MCBP modules; (2) the single-MCBP structure, where the MCBP module is only adopted to integrate the features from different domains, while there is no attention mechanism added. The structure of the single-MCBP based method is shown as follows. Compared with the proposed double-MCBP based method, the attention-based mechanism is removed and the fused textual and visual vectors are directly adopted for the detection. The structure of the single-MCBP based method is shown in Figure 5.

It can be seen from the table that the proposed double-MCBP module method is better than the single-MCBP method for the two datasets in terms of accuracy, precision, recall, and F1 score. Thus, the proposed double-MCBP module method shows better performance than the single-MCBP module method.

**Figure 6:**
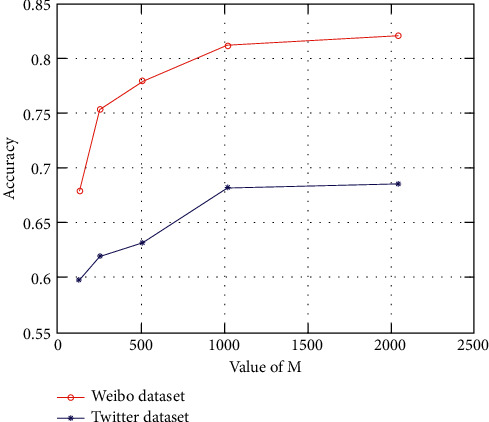
Different accuracies with different values of M for the Weibo and Twitter datasets.

In addition, in the proposed method, the number of feature dimensions adopted for feature fusion also has an important influence on the performance. Here, the number of feature dimensions adopted for fusion is denoted as *M*.  Figure 6 shows the changes of accuracies when *M* takes different values for the Weibo and Twitter datasets. The influences of different values of *M* are similar to the Weibo dataset. For the Twitter dataset, it can be seen that as the value of *M* increases from 128 to 1024, the accuracy has increased significantly. Compared with the situations when *M* equals 128, the accuracy has increased by 14.0%. When *M* equals 2048, compared with the situation when *M* equals 1024, accuracy has only increased less than 1%, which is very insignificant. For the Weibo dataset, it can be seen that as the value of *M* increases from 128 to 1024, the accuracy has increased significantly. Compared with the situations when *M* equals 128, the accuracy has increased by 19.8%. When *M* equals 2048, compared with the situation when *M* equals 1024, accuracy has only increased by about 1%, which is very insignificant. For the two conditions when *M* = 2048 and *M* = 1024, as the parameter size has expanded more than twice, the computational cost needed is also more than twice. Therefore, under the condition that for both the Weibo and Twitter datasets, the increased accuracy is less than 1%, the parameter *M* is valued as 1024.

### 3.2. Comparisons of Different Methods

In order to illustrate the effectiveness of the proposed method, comparisons with two other methods are made, which can be seen in Table 3. The comparison methods are the EANN method and the SpotFake method. The two methods are selected herein due to the following factors: (1) the proposed method, the EANN method, and the SpotFake method are all multimodal-based methods, which have adopted both textual and visual information for fake information detection; (2) the EANN method and the SpotFake method have adopted direct vector catenation for feature fusion, while the proposed method has adopted the MCBP module to improve feature fusion. The comparison results of the three methods under the Twitter dataset and the Weibo dataset are shown in the following table.

It can be seen that the method proposed is better than the EANN method and the SpotFake method in terms of accuracy, precision, recall, and F1 score. This can fully illustrate that the proposed method in this article can better integrate the features from different domains and has a better performance in fake information detection than methods adopting direct catenation of features from different domains.

## 4. Conclusions

With the widespread participation of users in OSN, fake information has also been widely spread on the social network and has had a deep impact on social development. Therefore, fake information detection was of great significance at the time when OSN is popularized. A fake information detection method adopting the MCBP module is proposed in this article. In the proposed method, the pretrained Bert model is adopted to obtain the textual representation vector, and the VGG-19 model is adopted to obtain the visual representation vector. The mentioned MCBP module is adopted twice. For the first time, the MCBP module is used to fuse textual representation vector and visual representation vector to obtain the visual representation vector with attention. Then, the visual representation vector with attention is fused with the textual representation vector to obtain a hybrid representation vector for the classification of true and fake information. The proposed method in this article is compared with the two baseline methods. The results on the Twitter and Weibo open-source datasets prove that the proposed method in this article is better than the EANN method and the SpotFake method in terms of accuracy, precision, recall, and F1 score.

## Figures and Tables

**Table 1:** The details for the Twitter dataset and the Weibo dataset.

	The Twitter dataset	The Weibo dataset
Fake	7898	4749
True	6026	4779
Total	13924	9528

**Table 2:** The accuracy, precision, recall, and F1 score comparisons under different datasets and under different network settings.

Dataset	Structure	Accuracy	Precision	Recall	F1 score
*Twitter*	Double-MCBP	0.682	0.596	0.830	0.693
	Single-MCBP	0.551	0.487	0.691	0.571

*Weibo*	Double-MCBP	0.812	0.833	0.781	0.806
	Single-MCBP	0.633	0.633	0.642	0.637

**Table 3:** The performance comparisons of the proposed method, the EANN method, and the SpotFake method.

Dataset	Method	Accuracy	Precision	Recall	F1 score
*Twitter*	EANN	0.623	0.547	0.747	0.631
	SpotFake	0.627	0.550	0.767	0.641
	Proposed	0.682	0.596	0.830	0.693

*Weibo*	EANN	0.727	0.736	0.711	0.723
	SpotFake	0.769	0.779	0.753	0.766
	Proposed	0.812	0.833	0.781	0.806

## Data Availability

The datasets used to support the findings of this study are available upon request to the author.
